# Pathogenic mutations in *NUBPL* affect complex I activity and cold tolerance in the yeast model *Yarrowia lipolytica*

**DOI:** 10.1093/hmg/ddy247

**Published:** 2018-07-04

**Authors:** Andrew E Maclean, Virginia E Kimonis, Janneke Balk

**Affiliations:** 1 Department of Biological Chemistry, John Innes Centre, Norwich NR4 7UH, UK; 2 School of Biological Sciences, University of East Anglia, Norwich NR4 7TJ, UK; 3 Division of Genetics and Genomic Medicine, Department of Pediatrics, University of California, Irvine, USA; 4 Children’s Hospital of Orange County, Orange, CA, USA

## Abstract

Complex I deficiency is a common cause of mitochondrial disease, resulting from mutations in genes encoding structural subunits, assembly factors or defects in mitochondrial gene expression. Advances in genetic diagnostics and sequencing have led to identification of several variants in *NUBPL* (nucleotide binding protein-like), encoding an assembly factor of complex I, which are potentially pathogenic. To help assign pathogenicity and learn more about the function of NUBPL, amino acid substitutions were recreated in the homologous Ind1 protein of the yeast model *Yarrowia lipolytica*. Leu102Pro destabilized the Ind1 protein, leading to a null-mutant phenotype. Asp103Tyr, Leu191Phe and Gly285Cys affected complex I assembly to varying degrees, whereas Gly136Asp substitution in Ind1 did not impact on complex I levels nor dNADH:ubiquinone activity. Blue-native polyacrylamide gel electrophoresis and immunolabelling of the structural subunits NUBM and NUCM revealed that all Ind1 variants accumulated a Q module intermediate of complex I. In the Ind1 Asp103Tyr variant, the matrix arm intermediate was virtually absent, indicating a dominant effect. Dysfunction of Ind1, but not absence of complex I, rendered *Y. lipolytica* sensitive to cold. The Ind1 Gly285Cys variant was able to support complex I assembly at 28°C, but not at 10°C. Our results indicate that Ind1 is required for progression of assembly from the Q module to the full matrix arm. Cold sensitivity could be developed as a phenotype assay to demonstrate pathogenicity of *NUBPL* mutations and other complex I defects.

## Introduction

Defects in respiratory chain function underlie a large share of mitochondrial disorders, of which a third are due to complex I deficiency (OMIM 252010) (1–3). Clinical presentations such as Leigh syndrome usually occur in infancy or early adulthood. Symptoms include skeletal muscle myopathy, cardiomyopathy, hypotonia, stroke, ataxia and lactic acidosis (3,4). Human complex I has 44 different structural subunits, of which 37 are encoded in the nuclear genome and 7 in the mitochondrial genome (5). Disease-causing mutations have been identified in 27 out of 44 structural genes. However, this only provides a diagnosis for about 50% of cases, as complex I deficiency may also be caused by mutations in assembly factors, tRNAs and other genes for mitochondrial gene expression or mitochondrial DNA maintenance (2,6). Assembly factors are defined as proteins which are required for the correct assembly and function of complex I but are not present in the mature structure. So far, 16 assembly proteins have been identified for complex I, and disease-causing mutations have been found in 11 of those (3,7). Particularly, advances in genome sequencing have led to the identification of several novel genes affecting complex I function, providing a genetic diagnosis for patients with these rare inheritable conditions.

The complex I assembly factor NUBPL (nucleotide binding protein-like) was first identified in the aerobic yeast *Yarrowia lipolytica* where it was named Ind1 (iron–sulfur protein required for NADH dehydrogenase) (8). Genomic deletion of *IND1* resulted in a major decrease in complex I, to approximately 28% of wild-type levels, but no effect on other respiratory complexes. Ind1 has been proposed to insert iron–sulfur (FeS) clusters in complex I based on its homology to the cytosolic FeS cluster assembly factors Nbp35 and Cfd1, corresponding to NUBP1 and NUBP2, respectively, in human (9). However, it should be noted that Ind1 is not essentially required for complex I in *Y. lipolytica*, and it would therefore only have an auxiliary role in cofactor assembly.

Phylogenetic analysis showed that the *IND1* gene is present in almost all eukaryotes, and closely matches the distribution of complex I (8). RNAi knockdown of *NUBPL* in human HeLa cells led to a specific decrease in complex I, as well as accumulation of assembly intermediates (10). Moreover, mutations in *NUBPL* were identified by exome sequencing in clinically described cases of complex I deficiency (1,11,12). Recently, *NUBPL* was found among 191 genes that are essential for oxidative phosphorylation in a genome-wide CRISPR/Cas9 screen of human leukemia K562 cells (13). The *IND1* homologue in the model plant *Arabidopsis thaliana* is also required for complex I assembly (14). Combined, these studies show that NUBPL has an evolutionary conserved function in complex I assembly, but its precise molecular function remains to be demonstrated.

So far, 13 cases (in 11 families) with likely clinically relevant mutations in *NUBPL* have been reported or newly diagnosed (Table 1). Patients display various symptoms within the broad spectrum of mitochondrial disorders, including motor problems, increased lactate levels and some degree of complex I deficiency (1,11,12). In addition, NUBPL patients show a distinct magnetic resonance imaging pattern with abnormalities of the cerebellar cortex, deep cerebral white matter and corpus callosum (11). Genetic analyses revealed that all but one patient have inherited an intronic mutation in one copy of *NUBPL*, c.815-27T>C, together with a likely deleterious mutation in the other copy of the gene (Table 1). The c.815-27T>C mutation affects a splicing branch site in intron 9, leading to aberrant mRNA splicing (15). Approximately 30% of wild-type *NUBPL* transcript remains, but two mis-spliced transcripts are also produced. One of these disappears by nonsense mediated decay. The other mis-spliced transcript lacks exon 10, leading to Asp273Gln, a frameshift and then a premature stop codon. The predicted protein product, p.(D273Qfs*31), is unstable, and NUBPL protein is almost undetectable in patient fibroblasts (15). The c.815-27T>C variant is often co-inherited with the missense variant c.G166>A (p.Gly56Arg), which, on its own, is not thought to be pathogenic (15), and is absent in at least two cases (Table 1, patient 2 and siblings 10 and 11). Recently, a clinical case of hereditary bilateral striatal necrosis was potentially attributed to biallelic missense variants in *NUBPL*, but neither the branch site mutation nor c.G166>A was found (Table 1, patient 9). It was suggested that this case represents the less severe end of the spectrum of NUBPL dysfunction, compared with the reported cases with the branch site mutation (16).

**Table 1:** Overview of mutations in *NUBPL* (NG_028349.1) associated with complex I deficiency or mitochondrial disease (OMIM 252010)

**Patient**	**Coding DNA**	**Protein**	**Site**	**Paternal or maternal**	**Reference**	
1	c.166G>A	p.(Gly56Arg)	Exon 2	Paternal	(1,15)	
	c.815-27T>C^a^	p.(Asp273Glnfs*31)	Intron 9	Paternal		
	240 kb deletion		Exons 1–4	Maternal		
	137 kb duplication		Exon 7	Maternal		
2	c.815-27T>C	p.(Asp273Glnfs*31)	Intron 9	Paternal	(12)	
	c.205_206delGT	p.(Val69Tyrfs*80)	Exon 2	Maternal		
3^b^	c.166G>A	p.(Gly56Arg)	Exon 2	N/A	(11)	
	c.815-27T>C	p.(Asp273Glnfs*31)	Intron 9	N/A		
4	c.166G>A	p.(Gly56Arg)	Exon 2	Paternal	(11)	
	c.815-27T>C	p.(Asp273Glnfs*31)	Intron 9	Paternal		
	c.667_668insCCTTGTGCTG	p.(Glu223Aspfs*4)	Exon 8	Maternal	
5,6	c.166G>A	p.(Gly56Arg)	Exon 2	Paternal	(11)	
	c.815-27T>C	p.(Asp273Glnfs*31)	Intron 9	Paternal	
	c.313G>T	p.(Asp105Tyr)	Exon 4	Maternal		
7	c.166G>A	p.(Gly56Arg)	Exon 2	Paternal	(11)	
	c.815-27T>C	p.(Asp273Glnfs*31)	Intron 9	Paternal		
	c.693+1G>A	p.?	Intron 8	Maternal		
8	c.579A>C	p.(Leu193Phe)	Exon 7	Paternal	(11)	
	c.166G>A	p.(Gly56Arg)	Exon 2	Maternal		
	c.815-27T>C	p.(Asp273Glnfs*31)	Intron 9	Maternal			
9	c.311T>C	p.(Leu104Pro)	Exon 4	Paternal	(16)	
	c.287A>T	p.(Asp96Val)	Exon 3	Maternal		
10, 11	c.815-27T>C	p.(Asp273Glnfs*31)	Intron 9	Paternal	Unpublished clinical cases^c^
	c.311T>C	p.(Leu104Pro)	Exon 4	Maternal	
12	c.166G>A	p.(Gly56Arg)	Exon 2	Maternal	Unpublished clinical case^c^	
	c.815-27T>C	p.(Asp273Glnfs*31)	Intron 9	Maternal	
	c.311T>C	p.(Leu104Pro)	Exon 4	Paternal		
13	c.166G>A	p.(Gly56Arg)	Exon 2	N/A	Unpublished clinical case^c^	
	c.815-27T>C	p.(Asp273Glnfs*31)	Intron 9	N/A	
	c.859G>T	p.(Gly287Cys)	Exon 10	N/A	

^a^The paternal c.166G>A mutation and the maternal exon deletions/duplication were identified by exome sequencing and a microarray, respectively (1), but further analysis identified the c.815-27T>C splice site mutation in the paternal allele (15).

^b^Kevelam et al. (11) could not confirm whether the mutations were homozygous or hemizygous because no parental DNA or fibroblasts were available for study. However, given the other cases, it is possible that another mutation in *NUBPL* is present to give a compound heterozygous situation.

^c^Clinical features for complex-I-deficiency patients 10, 11 and 12 have not been published (V. Kimonis, personal communication), but the mutations have been previously reported (41,42). Clinical features and mutations for patient 13 have not been published (H. Prokisch, personal communication).

The haploid yeast *Y. lipolytica* has been used extensively in biotechnology, but it has also been developed as a model organism to study complex I biology (17–21). This has been necessary because the usual yeast model Baker’s yeast, *Saccharomyces cerevisae*, does not have complex I. The D273Qfs*31 variant of NUBPL was recreated in the Ind1 protein of *Y. lipolytica*, which confirmed that the altered protein was unable to support the assembly of complex I (22). Here, we compare five missense variants in the *NUBPL* gene, two of which are newly identified in patients, in an effort to assign pathogenicity and to enhance our understanding of the molecular function of NUBPL/Ind1. The resulting protein variants were tested in *Y. lipolytica* for protein stability and complex I levels, oxidoreductase activity and assembly intermediates. Together, the results indicate pathogenicity of four missense mutations and reveal that *ind1* mutants are sensitive to low temperature.

## Results

### Selection of *NUBPL* mutations for analysis in *Y. lipolytica*

Thirteen cases of mitochondrial disorders associated with mutations in *NUBPL* have been published or were otherwise known to us (Table 1). Mutations that result in frameshifts, insertions or deletions (patients 1–4 and 7) are almost certainly deleterious but are unlikely to shed light on the molecular function of NUBPL. Instead, we focused on assessing the functional impact of patient mutations resulting in a single amino acid change at a conserved position, such as Leu104Pro (L104P, patients 9, 10, 11 and 12), Asp105Tyr (D105Y, patients 5 and 6), Leu193Phe (L193F, patient 8) and Gly287Cys (G287C, patient 13). Many more missense variants in *NUBPL* have been uncovered by large-scale exome sequencing (gnomad.broadinstitute.org), of which Asn198Tyr and Val182Ala occur at a relatively high allele frequency (Table 2). While Asn198 and Val182 are not conserved in *Y. lipolytica* Ind1, the NUBPL Gly138Asp (G138D) variant with an allele frequency of 6.73 × 10^-4^ does affect a conserved amino acid, corresponding to Gly136 in *Y. lipolytica* Ind1, and was included in this study. G138D is predicted to be damaging to NUBPL (Table 2) and has been found in two Parkinson’s disease patients (P. Eis and E. Hatchwell, personal communication).

**Table 2:** Allele frequencies and PolyPhen-2 scores of NUBPL missense variants

Protein variant	1-letter code	Allele count	Total alleles	Allele frequency	Conserved in *Y. lip*	PolyPhen-2
p.(Asn198Thr)	N198T	1207	276746	4.36 × 10^-3^	No	0.888
p.(Asp273Glnfs*31)		1000	276338	3.62 × 10^-3^	N/A	N/A
due to c.815-27T>C						
p.(Val182Ala)	V182A	730	276900	2.64 × 10^-3^	No	0.923
p.(Gly26Val)	G26V	265	149738	1.77 × 10^-3^	No	0.041
p.(His229Tyr)	H229Y	349	276920	1.26 × 10^-3^	No	0.754
p.(Gly138Asp)	G138D	185	275070	6.73 × 10^-4^	Yes	0.999
p.(Ser128Asn)	S128N	57	264100	2.16 × 10^-4^	No	0.000
p.(Leu104Pro)	L104P	40	276904	1.45 × 10^-4^	Yes	1.000
p.(Lys59Arg)	K59R	39	276958	1.41 × 10^-4^	No	0.187
p.(Gly56Arg)	G56R	38	276640	1.37 × 10^-4^	No	0.998
p.(Asp105Tyr)	D105Y	6	276922	2.17 × 10^-5^	Yes	1.000
p.(Asp96Val)	D96V	N/A	N/A	N/A	No	0.714
p.(Leu193Phe)	L193F	N/A	N/A	N/A	Yes	1.000
p.(Gly287Cys)	G287C	N/A	N/A	N/A	Yes	1.000

Allele frequency data from gnomAD (http://gnomad.broadinstitute.org/) for the most commonly occurring missense mutations in *NUBPL*, as well as the clinically relevant variants listed in Table 1. A PolyPhen-2 score close to 1 indicates that the amino acid change is “probably damaging”.

**Figure 1:**
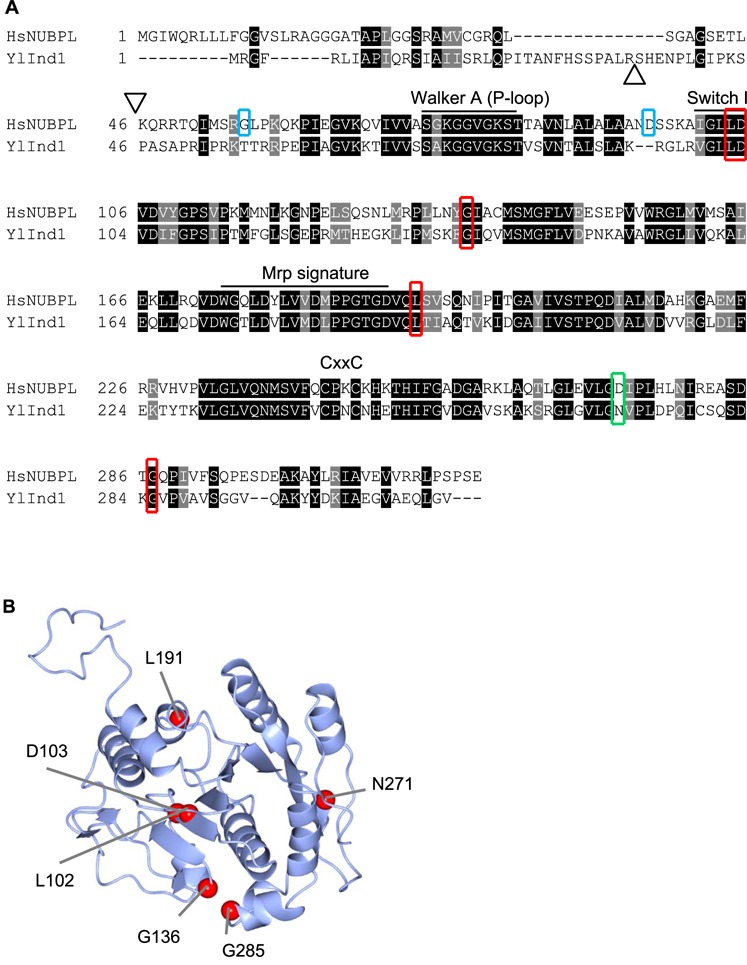
Amino acid substitutions in NUBPL and their position in the homologous Ind1 protein in *Y. lipolytica*. (**A**) Alignment of amino acid sequences of human (Hs) NUBPL (NP_079428.2) and *Y. lipolytica* (Yl) Ind1 (XP_501064.1) using Clustal Omega and Boxshade for colouring. Amino acid changes that are investigated in this study are indicated with red boxes. Amino acid changes associated with clinical cases but which are not conserved in *Yarrowia* are indicated with blue boxes. Positions D273 in NUBPL and N271 in Ind1 are indicated with a green box. The start of the mature N-terminus is indicated with a triangle. The Walker A motif (P-loop), Switch I, Mrp family signature (ProSite entry PS01215) and CxxC motif are indicated. (**B**) Homology model of Ind1 using IntFOLD server using template 3vx3A.pdb (HypB from *Thermococcus kodakarensis* KOD1). Amino acid residues that were substituted in this study are indicated with red spheres.

L104P and D105Y affect residues in the highly conserved Switch I motif (Fig. 1A), which is involved in ATP hydrolysis; L193F affects a residue just outside the Mrp family signature motif; and G287C introduces an additional cysteine to the C-terminus, which may create an unwanted disulphide bridge. G138 is not in a conserved motif, but the introduction of a negatively charged Asp could affect protein folding. The corresponding amino acids in the Ind1 protein of *Y. lipolytica* were identified by alignment using Clustal Omega (Fig. 1A; Table 3), and this amino acid numbering is used throughout the study. A homology model of Ind1 was made, and the positions of the substituted amino acids are indicated in Fig. 1B.

### Protein stability in Ind1 variants

In order to study the biochemical and physiological effects of the amino acid changes, site-directed mutations were introduced into a plasmid carrying the *IND1* gene under the control of its native promoter and transformed into *Y. lipolytica ind1*Δ cells, a GB10 strain with a genomic deletion of the *IND1* gene (8). The plasmid contains a chromosomal ARS/CEN region which is maintained at ∼1 copy per cell. The GB10 strain was engineered to contain the *NDH2i* gene, encoding an alternative NADH dehydrogenase targeted to the matrix side of the inner mitochondrial membrane, which bypasses the essential requirement of respiratory complex I in *Y. lipolytica* (23). The previously reported variant Ind1 protein N271Qfs*31, recapitulating the effect of the c.815-27T>C branch-site mutation in *NUBPL*, was included for comparison (22).

**Figure 2:**
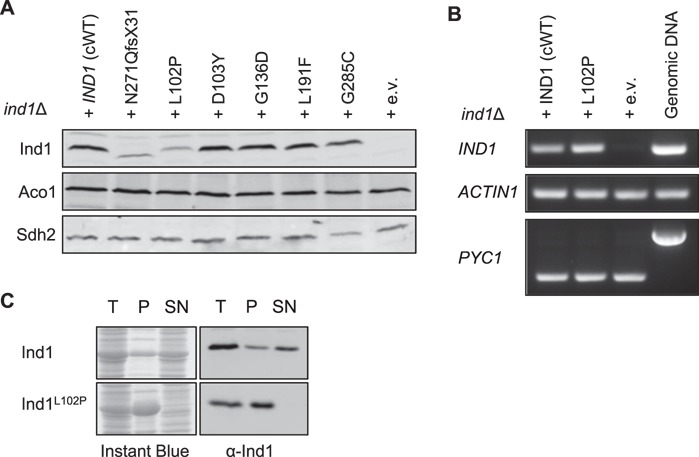
Expression of variant Ind1 proteins in *Y. lipolytica.* (**A**) Protein blot analysis of Ind1 in mitochondrial membranes from strains expressing wild-type *IND1* (cWT) and the indicated protein variants, in the *ind1*Δ background. The N271Qfs*31 protein is of lower molecular weight due to truncation of the C-terminus by 11 amino acids. Antibodies against the mitochondrial protein aconitase (Aco1) and subunit 2 of succinate dehydrogenase (Sdh2) were used to confirm equal protein loading and stability of these FeS cluster proteins in *ind1* mutants. (**B**) Transcript levels of *IND1*^L102P^ compared with wild-type *IND1* by RT-PCR. A PCR reaction for *PYC1* (*YALI0C24101g*), containing an intron, showed that the cDNA samples were free from genomic DNA and equal in cDNA content. *ACTIN1* was used as an additional control for equal amounts of cDNA template. (**C**) Solubility of wild-type Ind1 and Ind1^L102P^ protein expressed in *E. coli*. Total protein extract (T) was sonicated and centrifuged to give insoluble pellet (P) and soluble supernatant (SN). Gels were stained with Instant Blue (left panels) or immunolabelled for Ind1 (right panels).

Mitochondrial membranes of each strain were isolated and subjected to Western blot analysis to visualize Ind1 protein levels. The D103Y, G136D, L191F and G285C variants displayed levels of Ind1 protein similar to *ind1*Δ + *IND1* (henceforth referred to as complemented wild type, cWT) (Fig. 2A). The L102P substitution resulted in lower levels of Ind1 protein, to approximately 45% of cWT levels. The decrease in L102P protein was similar to that seen in N271Qfs*31. Antibodies against aconitase (Aco1) and subunit 2 of complex II (Sdh2) were used as a loading control and to confirm that other FeS cluster binding proteins are not affected (Fig. 2A).

**Table 3:** Summary of human and *Yarrowia* variants analyzed in this study

*H. sapiens* amino acid variant	*Y. lipolytica* amino acid variant	Protein stability	Complex I levels	Assembly intermediates	Growth in cold
L104P	L102P	Decreased	Severely decreased	NUBM and NUCM	Impaired
D105Y	D103Y	Normal	Severely decreased	NUBM and NUCM	Impaired
G138D	G136D	Normal	Normal	NUCM	Normal
L193F	L191F	Normal	Slightly decreased	NUCM	Impaired
G287C	G285C	Normal	Normal	NUCM	Impaired
D273Qfs*31	N271Qfs*31	Decreased	Severely decreased	NUBM and NUCM	Impaired

To rule out that lower amounts of the Ind1^L102P^ protein were due to decreased transcript levels, expression of *IND1* was assessed by reverse transcription–polymerase chain reaction (RT-PCR) (Fig. 2B). While this technique is not quantitative, at low cycle numbers, major differences in transcript levels are easily detected. Normal levels of *IND1* transcript in the L102P variant indicated that the protein undergoes post-translational degradation. In order to further investigate the effect of L102P on protein stability, Ind1 and Ind1^L102P^ were expressed in *E. coli*. Separation of soluble and insoluble fractions showed that all of the Ind1^L102P^ protein is found in the insoluble pellet fraction, whilst most of the normal Ind1 protein can be found in the soluble fraction. This suggests that the L102P substitution causes protein misfolding, which would result in its degradation by proteases. Overall these data show that, with the exception of L102P, the selected amino acid substitutions in Ind1 do not affect protein stability.

### Ind1 variants L102P, D103Y and L191F have significantly decreased levels of complex I

**Figure 3:**
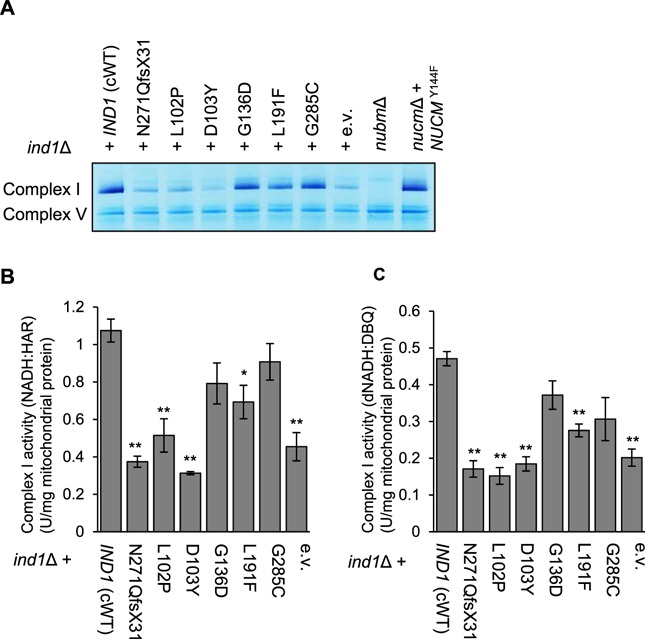
Complex I levels and oxidoreductase activities in Ind1 variants.(**A**) Complex I levels shown by in-gel NADH:NBT staining of mitochondrial membranes from *ind1*Δ producing wild-type *IND1* (cWT) and the indicated protein variants, compared with two characterized complex I mutants, *nubm*Δ and *nucm*Δ + *NUCM*^Y144F^. (**B**) NADH:HAR and (**C**) dNADH:DBQ oxidoreductase activity in mitochondrial membranes from the indicated strains. Bars represent the mean ± SE (*n* = 3 biological replicates). * *p*<0.05, ** *p*<0.01 (two-sample *t*-test). Numerical values are given in Table S1.

It has previously been shown that cells lacking *IND1* have approximately 28% of fully active complex I compared with wild type (8). To investigate the effect of the amino acid variants in Ind1 on complex I, mitochondrial membranes were separated by blue-native polyacrylamide gel electrophoresis (BN-PAGE) to resolve the respiratory complexes. Incubation of the gel with NADH and nitro-blue tetrazolium (NBT) reveals NADH dehydrogenase activities, which is used as a proxy to estimate complex I levels (24). In cells expressing the Ind1 variants L102P and D103Y, complex I was strongly decreased, similar to the levels in the *ind1*Δ mutant (empty vector control, e.v.) and N271Qfs*31 (Fig. 3A). The L191F substitution resulted in a minor decrease in complex I, whereas G136D and G285C were indistinguishable from cWT. For comparison, a knockout strain of the NUBM subunit of complex I (*nubm*Δ) displayed no complex I activity, in agreement with the FMN cofactor of NUBM being the site of NADH oxidation. Another complex I mutant, where a key catalytic residue of the NUCM subunit has been substituted (Y144F) (25), displayed complex I levels similar to cWT. This is consistent with a mutation that affects catalysis but not assembly of complex I. Complex V levels were unaffected by mutations in *IND1* (Fig. 3A, lower Coomassie-stained band).

To measure the oxidoreductase activity of the matrix arm of complex I, two different spectrophotometric assays were used. Electron transfer from NADH to the artificial electron acceptor hexaammineruthenium(III) chloride (HAR) involves only the primary electron acceptor FMN bound to the NUBM subunit of complex I and, like NADH:NBT staining, serves as a proxy for complex I content. The L102P, D103Y, L191F and N271Qfs*31 variants showed a significant decrease in NADH:HAR activity, whereas G136D and G285C were similar to cWT (Fig. 3B, Table S1).

To assess NADH:ubiquinone oxidoreductase activity, electron transfer from deamino-NADH (dNADH) to n-decylubiquinone (DBQ) was measured, encompassing all cofactors in the matrix arm of complex I. A significant decrease in electron transfer was seen with the L102P, D103Y, L191F and N271Qfs*31 variants, but not in G136D and G285C (Fig. 3C, Table S1). The *nucm*Δ + *NUCM*^Y144F^ mutant had low dNADH:DBQ activity (Table S1), despite wild-type levels of fully assembled complex I (Fig. 3A). Comparison of dNADH:DBQ activity between the Ind1 variants and *nucm*Δ + *NUCM*^Y144F^ shows that in the Ind1 variants there is still substantial activity remaining. For the Ind1 variants, the NADH:HAR activities correspond with those of dNADH:DBQ, suggesting that all the complex I present is enzymatically active.

Taken together, the data from three different methods are in agreement and indicate that the L102P and D103Y substitutions in Ind1 cause a defect in complex I assembly of similar severity as *IND1* deletion. The L191F substitution caused a mild decrease, whereas G136D and G285C appeared to have no significant effect on complex I levels under standard growth conditions.

### All *ind1* mutants accumulate a Q module assembly intermediate

**Figure 4:**
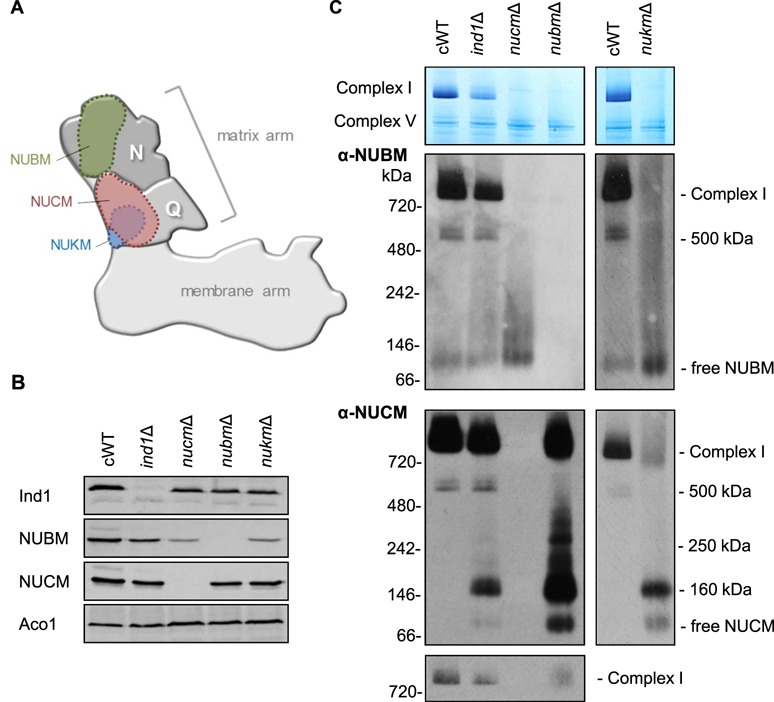
Complex I assembly in *ind1*Δ compared with deletion mutants of other subunits.(**A**) Diagram of complex I showing the position of NUBM in the N module and NUCM and NUKM in the Q module of the matrix arm. (**B**) Mitochondrial membranes from the indicated complex I strains (complemented wild type [cWT], *ind1*Δ, *nucm*Δ, *nubm*Δ and *nukm*Δ) were separated by SDS-PAGE. Western blots were labelled with antibodies against Ind1 and the complex I subunits NUBM and NUCM, as indicated. Antibodies against mitochondrial aconitase, Aco1, were used to confirm equal loading and protein transfer. (**C**) Mitochondrial membranes were separated by BN-PAGE. Complex I is shown by in-gel NADH:NBT staining (top panel) and by Western blot analysis with antibodies against NUBM and NUCM. The immunoblots were exposed for a relatively long time to visualize all assembly intermediates, but short exposure of the NUCM signal of fully assembled complex I is also shown (bottom panel).

In human cell lines depleted of *NUBPL*, a subcomplex representing part of the membrane arm was observed (10). Similarly, the Arabidopsis *indh* mutant had a membrane arm assembly intermediate, but no full-size complex I (14). To investigate complex I assembly in *Y. lipolytica* cells lacking Ind1, the *ind1*Δ strain was compared with mutant strains of different subunits of the matrix arm of complex I, *nubm*Δ, *nucm*Δ and *nukm*Δ. NUBM is the homologue of the human NDUFV1 protein (bovine 51-kDa subunit) in the N module. NUCM (human NDUFS2, bovine 49 kDa) and NUKM (human NDUFS7, bovine PSST) are in the Q module (Fig. 4A). In the *ind1*Δ mutant, the protein levels of NUBM are slightly decreased, but the levels of NUCM are not affected (Fig. 4B). Thus, compared with the decreased levels of complex I in *ind1*Δ, NUBM and NUCM are imported into the mitochondrial matrix, but the majority is not assembled. Decreased levels of NUBM are also seen in the *nucm*∆ and *nukm*∆ mutants.

Mitochondrial membranes were isolated from cWT, *ind1*Δ, *nubm*Δ, *nucm*Δ and *nukm*Δ, and the respiratory complexes were separated by BN-PAGE, followed by in-gel activity staining and Western blot analysis with antibodies against NUBM and NUCM. This confirmed the lack of complex I in the *nucm*Δ and *nukm*Δ mutants (Fig. 4C). In the *nubm*Δ mutant, NUCM antibodies detected a weak signal around ∼1 MD (Fig. 4C, bottom panel), likely corresponding to a small amount of complex that lacks NUBM. NADH:NBT activity staining did not detect any full-size complex I in *nubm*Δ, consistent with NUBM being the site of NADH oxidation. At longer exposure times to visualize assembly intermediates, a subcomplex of approximately 500 kDa containing both NUBM and NUCM was seen in the *ind1*Δ mutant, which was also found in cWT (Fig. 4C). This subcomplex is likely to represent the full matrix arm of complex I (26). An additional assembly intermediate containing NUCM, but not NUBM, was found at approximately 160 kDa. This intermediate can also be seen in *nubm*Δ and *nukm*Δ, but not in cWT, and likely corresponds to the Q module. Kmita et al. (24) have previously reported a Q module assembly intermediate in *Y. lipolytica* containing the NUCM, NUGM, NUKM and NUFM subunits. Combined, these subunits would have a molecular weight of 116.42 kDa (calculated based on molecular weights in Angerer et al. (28)). Assembly factors would likely also be bound to this structure, resulting in the observed molecular weight of ∼160 kDa. These data show that while mitochondrial membranes of *ind1*Δ cells have some fully assembled complex I and a 500 kDa intermediate, there is accumulation of a Q module intermediate.

**Figure 5:**
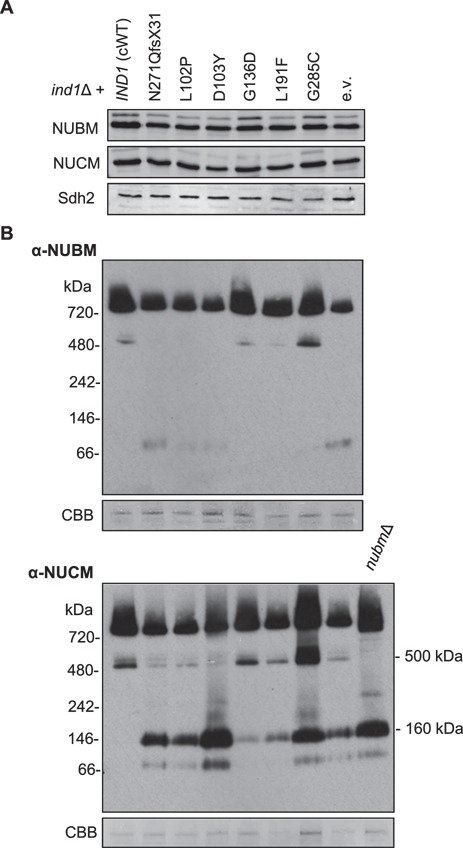
Complex I assembly intermediates in Ind1 variants. Mitochondrial membranes from *ind1*Δ cells expressing wild-type *IND1* (cWT) and the indicated protein variants were separated by SDS-PAGE (**A**) or by BN-PAGE (**B**). The gels were blotted and labelled with antibodies against NUBM, NUCM or Sdh2. CBB, Coomassie Brilliant Blue staining of complex V was used as a loading control.

Next, we investigated the assembly of complex I in the Ind1 protein variants. Interestingly, the G136D and G285C variants that have normal complex I levels (Fig. 3A) visibly accumulated the Q module intermediate containing NUCM (Fig. 5B, lower panel). Generally, the pattern of assembly intermediates containing NUCM is similar in all mutants, except that Ind1^D103Y^ accumulated relatively more Q module intermediate and very little of the 500 kDa intermediate. For intermediates containing the NUBM subunit, variants with low levels of complex I (N271Qfs*31, L102P and D103Y) closely resembled the empty vector control, whereas those with near normal complex I levels (G136D, L191F, G285C) more closely resemble cWT (Fig. 5B, upper panel).

### *ind1* mutants are sensitive to cold

**Figure 6:**
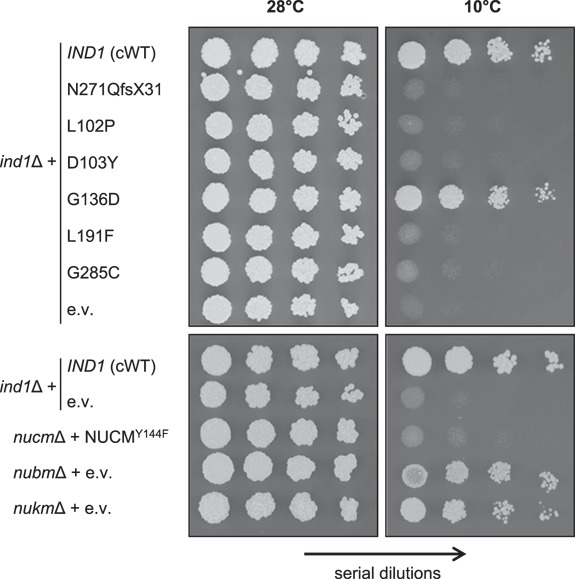
*ind1* mutants are sensitive to low temperature. Growth of *ind1*Δ cells expressing wild-type *IND1* (cWT) and the indicated protein variants, and complex I mutants *nucm*Δ + *NUCM*^Y144F^, *nubm*Δ and *nukm*Δ grown at normal (28°C) and cold (10°C) temperatures. e.v., empty vector. Images show serial dilutions of cell cultures spotted onto agar plates.

During storage of *Y. lipolytica* strains, it was noticed that the *ind1*Δ mutant was sensitive to cold, exacerbating the mild growth defect. To investigate this further, the growth of *ind1* mutants, as well as *nucm*Δ + *NUCM*^Y144F^, *nubm*Δ and *nukm*Δ, was compared at 28°C and 10°C. Using a standard drop assay for yeast growth, cells were spotted onto agar plates in a dilution series. Each cell seeds a new colony, the diameter of which is determined by cell division rate and cell size. After incubation of the plates overnight at 28°C plates to initiate growth, they were placed either at 28°C for a further 2 days or moved to 10°C for 10–14 days. At 28°C all strains grew similarly, despite slight variations in colony size (Fig. 6, left panels). However, when grown at 10°C, some strains displayed a dramatic growth retardation (Fig. 6, right panels). The *ind1*Δ strain expressing wild-type *IND1*, Ind1 variant G136D, *nubm*Δ and *nukm*Δ all displayed significant growth at 10°C. However, all other Ind1 variants displayed very slow or no further growth at 10°C. Interestingly, the *nubm*Δ and *nukm*Δ mutants that lack complex I completely were able to grow in cold conditions, but *nucm*Δ + *NUCM*^Y144F^ with an inactive form of complex I could not grow, similar to the *ind1*Δ mutant.

These data reveal a novel growth difference of certain complex I mutants grown at normal (28°C) or cold (10°C) conditions. Ind1 variants, except for G136D, also display this temperature-dependent growth defect.

### Complex I assembly in the Ind1^G285C^ variant is temperature sensitive

**Figure 7:**
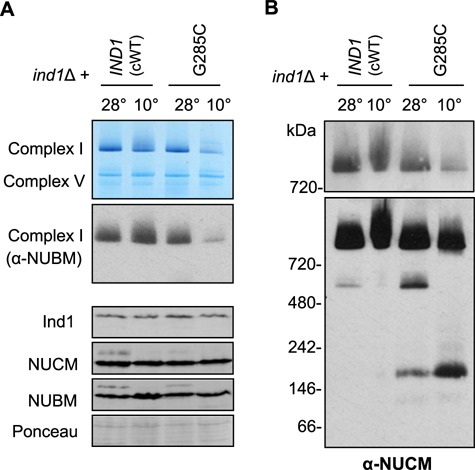
The Ind1 G285C variant cannot assemble complex I at low temperature. (**A**) Complex I levels in mitochondrial membranes from *ind1*Δ expressing wild-type *IND1* (cWT) or Ind1^G285C^ following growth at 28°C or 10°C. Complex I was visualized by BN-PAGE and NADH:NBT staining and by Western blotting with antibodies against NUBM. The levels of Ind1, NUCM and NUBM were assessed by standard SDS-PAGE and Western blot analysis. Ponceau S staining was used to confirm equal loading and transfer. (**B**) Complex I assembly intermediates containing the NUCM subunit from samples as in (A) were detected by Western blot analysis of BN-PAGE gels. Top panel, short exposure; bottom panel, long exposure.

The Ind1 variant G285C has normal levels and activity of complex I (Fig. 3A). However, it accumulated the Q module intermediate (Fig. 5B), and growth at 10°C was impaired (Fig. 6). To investigate if assembly of complex I in the G285C variant is conditional on temperature, mitochondrial membranes were isolated from cells producing wild-type Ind1 (cWT) and the Ind1 G285C variant after overnight growth at 10°C and compared with samples grown at 28°C. Mitochondrial membranes were separated by BN-PAGE and stained with NADH:NBT to assess formation of complex I. cWT displayed full complex I assembly in cultures grown at both 28°C and 10°C (Fig. 7A). By contrast, the G285C Ind1 variant could not support complex I assembly when grown at 10°C, with complex I levels decreased to those in the *ind1*Δ mutant (Fig. 3A). BN-PAGE followed by Western blotting and labelling with antibodies against NUBM confirmed the significant decrease in complex I (Fig. 7A). Immunodetection of Ind1 showed that the G285C protein variant is stable at 10°C, meaning that the decrease in complex I levels is not due to a cold-dependent degradation of the Ind1^G285C^ protein.

Next, the effect of cold conditions on assembly intermediates of complex I was assessed. Mitochondrial membranes were separated by BN-PAGE followed by Western blotting with antibodies against NUCM. A short exposure of the signal confirmed the decreased levels of fully assembled complex I in cells expressing the G285C Ind1 variant grown at 10°C (Fig. 7B, top panel). A longer exposure revealed a different pattern of complex I assembly intermediates in mitochondria isolated at normal and cold temperatures (Fig. 7B). Ind1^G285C^ samples from cold conditions lacked a band corresponding to the assembly intermediate at 500 kDa, made up of the N and Q modules, and an increase in the Q module intermediate at 160 kDa. This suggests that the G285C Ind1 variant accumulates greater levels of the Q module at cold temperatures, as it is unable to assemble it into the 500 kDa intermediate. There is still a strong signal at the size corresponding to complex I; however, this is due to a long exposure time needed to see assembly intermediates. Taken together, these data show that the Ind1 G285C variant is more severely impaired in complex I assembly when grown in cold conditions.

## Discussion

Since *NUBPL* was associated with mitochondrial disease resulting from complex I deficiency (1,11,12), the gene is routinely included in the list of candidate genes for genetic diagnoses. In children’s hospitals around the world, additional mutations have been found in *NUBPL* that are likely to be pathogenic. In this study five protein variants of human NUBPL were recreated in the homologous Ind1 protein in the yeast *Y. lipolytica* to study the effect of the amino acid changes on complex I assembly and to confirm pathogenicity.

Ind1 variants L102P and D103Y showed the strongest decrease in complex I, similar to the effect of N271Qfs*31 and deletion of the *IND1* gene (Fig. 3). The L102P substitution destabilized the Ind1 protein (Fig. 2), which most likely explains the complete loss of functionality. The D103Y substitution does not affect protein stability, suggesting that the amino acid change renders Ind1 inactive. Asp103 is situated at the N-terminal end of the Switch I motif (Fig. 1A). In molecular motors driven by ATP (or GTP) hydrolysis, the Switch I and II sequences form the nucleotide binding pocket, while a conserved lysine residue of the Walker A motif interacts with ATP (29). Upon ATP hydrolysis, Switch I and II undergo large conformational changes, facilitating monomer–dimer transitions (e.g. in ParA and MinD in bacterial cell division) or interactions with other proteins (e.g. NifH in nitrogenase). D103 aligns with D38 in MinD of *Escherichia coli,* which is the residue required for dimerization of MinD as well as for interaction between MinD and MinC (30). NUBPL and other members of the NBP35/Mrp subfamily of P-loop NTPases also form dimers (9). Lack of dimer formation or lack of interaction with another protein, for example a subunit of complex I, is likely to fully abolish the function of NUBPL/Ind1. Interestingly, the Ind1^D103Y^ variant appears to have a block in assembling the N module onto the Q module, as very little matrix arm assembly intermediate was observed and high levels of Q module accumulated (Fig. 5B). Possibly, a failure to use ATP hydrolysis for conformational changes of Ind1 may have a dominant effect and trap a specific assembly intermediate.

The Ind1^L191F^ variant caused a moderate decrease in complex I levels and activity. This residue is close to the Mrp family signature (Fig. 1A) found only in the subfamily of NBP35/Mrp P-loop NTPases. Members of this subfamily are found in archaea, bacteria and eukaryotes. The precise molecular function of this amino acid motif is not known.

The G136D substitution in Ind1 had no significant effect on complex I levels and redox activities (Fig. 3); however, the variant protein caused accumulation of the Q module assembly intermediate, albeit at low levels (Fig. 5). The equivalent G138D variant in human NUBPL has so far not been associated with a clinical case of complex I deficiency even though it is found at ∼5-fold higher frequency (Table 2) than the L104P variant (to date, the most frequent complex I deficiency missense mutation that has been found). Both G138D and c.815-27T>C variants have been observed in the heterozygous state in Parkinson’s disease patients (P. Eis and E. Hatchwell, personal communication), albeit not at a significantly higher frequency compared with publicly reported subjects (gnomad.broadinstitute.org). Since the allele frequency for G138D and c.815-27T>C are moderately high (Table 2), homozygotes or compound heterozygotes for these variants are likely to exist, and they may have late onset symptoms linked to mild complex I deficiency that are clinically relevant in diseases involving mitochondrial dysfunction, such as Parkinson’s disease, as suggested by others (15,31).

It is possible that in the case of ‘mild’ *IND1* mutations, effects on complex I assembly only manifest themselves under certain conditions, as for the Ind1 G285C variant. At the normal growth temperature of *Y. lipolytica* (28°C), there was no significant effect on complex I levels and activities, although accumulation of the Q module assembly intermediate was observed (Fig. 5). At low temperature (10°C), the Ind1 G285C variant showed a dramatic decrease in complex I and increased levels of the Q module intermediate (Fig. 7). Glycine 285 is located at the end of two short alpha helices (Fig. 1C), and a cysteine may disrupt this structure. Alternatively, the cysteine, if exposed, may form an unwanted disulfide bridge with another cysteine, in particular the cysteines of the CxxC motif, which are important for the function of NUBPL/Ind1 (8,10). A Gly to Cys substitution in Nfu1, a mitochondrial protein involved in FeS cluster assembly that also carries a CxxC motif, causes a dominant genetic effect in yeast (32) and severe mitochondrial disease in humans (33). Why the effects of G285C are exacerbated at cold temperatures remains an open question.

At the normal growth temperature of 28°C, *Y. lipolytica* containing Ind1 variants and complex I mutants grew similarly to cWT, because NADH oxidation in these strains is maintained by expression of ND2i, a single subunit NADH dehydrogenase on the matrix side. However, at 10°C, functionally compromised Ind1 variants and *nucm*∆ + *NUCM*^Y144F^ displayed almost no growth, whereas the *nubm*∆ and *nukm∆* strains were comparable with cWT in cold tolerance (Fig. 6). An initial hypothesis for this difference is that strains with a non-functional complex I produce large amounts of reactive oxygen species (ROS) such as superoxide (5), which may be exacerbated in the cold. Interestingly, lack of the accessory NDUFS4 subunit of complex I in the model plant Arabidopsis was associated with increased ROS production and diminished cold tolerance (34). The *nukm∆* mutant lacks complex I completely, and the *nubm*∆ lacks the FMN site where most ROS is produced. Further investigation is needed to uncover the cause of this growth defect.

In summary, of the five NUBPL missense variants that were functionally assessed in *Y. lipolytica*, four (L104P, D105Y, L193F and G285C) were found to be deleterious to complex I and one (G138D) was found to exhibit a mild defect. Along with other studies (18,21,35,36), these results reconfirm the utility of *Y. lipolytica* as a model for human mitochondrial disorders. However, care must be taken in over-interpreting the results, as some effects seen in *Y. lipolytica* have not been found in humans. In human *NUBPL* RNAi HeLa lines, the levels of NDUFV1 subunit are substantially decreased (10), whereas in the *Y. lipolytica ind1*∆ mutant, the levels of the homologous NUBM protein are only slightly less than in cWT. Secondly, the Ind1^D103Y^ variant appears to have stable protein levels in *Y. lipolytica*, but human patients with D105Y have almost no NUBPL protein (11). Ideally, all clinically relevant *NUBPL* mutations should also be tested in human cell lines, although *Y. lipolytica* is useful as a cost-effective model to study potentially pathogenic mutations affecting complex I.

A defect in Q module assembly in *Y. lipolytica ind1* mutants is consistent with studies of NUBPL in human cell lines (10,11). The Q module has 3 FeS clusters that may be specifically inserted by NUBPL. However, detailed proteomic analysis of complex I assembly intermediates did not find NUBPL associated with any subcomplexes; it only occurred as a dimer (37). Possibly, NUBPL interacts with an individual Q module subunit, or the protein interaction with the Q module assembly intermediate is transient. The Ind1 protein variants characterized in this study, in particular the D103Y variants, could serve as a tool to unravel exactly which step in complex I assembly is mediated by NUBPL/Ind1.

## Materials and Methods

### GenBank Accession Numbers

NUBPL, *Homo sapiens*: NG_028349.1; NP_079428.2

Ind1, *Yarrowia lipolytica*: XP_501064.1

### Yeast strains and growth

The *Y. lipolytica ind1*Δ strain in the GB10 genetic background (*ura3-302, ind1*::*URA3, leu2-270, lys11-23, NUGM-Htg2, NDH2i, MatB*) was used to analyze mutant versions of *IND1* and has been described previously (8). The *IND1* gene (*YALI0B18590g*) including its native promoter (1 kb upstream of the ATG) was reintroduced using the pUB4 plasmid (23) with a hygromycin selection marker, *HygB* from *Klebsiella pneumoniae*, to obtain the complemented wild type (cWT). The *nubm*Δ*, nucm*Δ, *nucm*Δ + NUCM^Y144F^ and *nukm*Δ were as previously described (18,25,38) but transformed with ‘empty’ pUB4 plasmid to grow the cells in the presence of hygromycin. *Y. lipolytica* cells were grown in rich medium containing 1% (w/v) yeast extract and 1% (w/v) glucose (Y$^{1}\!/_{\!2} $D), either in liquid culture or on solid medium with 2% (w/v) agar. Hygromycin B (75 μg ml^-1^) was added for selection of cells transformed with the pUB4 plasmid.

### Molecular cloning and mutagenesis

The *IND1* sequence from pUB4-*IND1-strep* (8) was cloned into the pGEM-T Easy vector (Promega, Madison, WI USA) and used for mutagenesis using a QuikChange® II kit (Agilent, Santa Clara CA, USA), as per the manufacturer’s instructions. Mutagenesis primers were designed using the online QuikChange primer design tool, hosted at http://www.genomics.agilent.com/primerDesignProgram.jsp (Table S2). Mutated *IND1*-strep sequence was inserted into pUB4, between XbaI and NsiI restriction sites. Plasmids were confirmed by sequencing and diagnostic restriction digest.

*Y. lipolytica* cells were transformed as described (39). Briefly, cells were grown overnight in YP$^{1}\!/_{\!2} $D (1% (w/v) yeast extract, 2% (w/v) peptone and 1% (w/v) glucose, at 28°C, and collected by centrifugation, or alternatively harvested from a fresh YP$^{1}\!/_{\!2} $D plate. Cells were resuspended in 100 μl buffer [45% (w/v) polyethylene glycol 4000, 0.1 M lithium acetate pH 6.0, 0.1 M dithiothreitol, 250 μg ml^-1^ single-stranded carrier DNA] and 200–500 ng plasmid was added. The mixture was incubated at 39°C for 1 h and then spread on a YP$^{1}\!/_{\!2} $D plate containing 75 μg ml^-1^ Hygromycin B. Transformants were visible after 2–3 days growth at 28°C.

### Recombinant protein expression

*IND1* sequences encoding the Ind1 variants L102P and D103Y, excluding the first 36 amino acids, were cloned from pUB4 into pET15b. Ind1-strep was expressed and purified as described in Bych et al. (8). Briefly, plasmids were transformed into *E. coli* Rosetta containing a plasmid with the genes for iron–sulfur cluster assembly (pISC) and plasmidpLYS. Colonies were grown overnight at 37°C in Lysogeny broth (LB) media, with required antibiotics, and then diluted 50× in 100 ml Terrific broth [47.6 g l^-1^ Teriffic broth, 0.8 ml 50% (v/v) glycerol] and grown until OD600 = 0.6. Protein expression was induced by addition of 1 ml l^-1^ benzylalcohol, 50 μm Fe–ammonium citrate, 100 μm L-cysteine and 1.2 ml l^-1^ isopropyl β-D-1-thiogalactopyranoside (IPTG). Cells were grown at 20°C overnight, then harvested by centrifugation at 5000 × *g* and flash frozen in liquid nitrogen. Cells were then resuspended in buffer W (100 mm Tris–HCl pH 8.0, 150 mm NaCl) with 0.2% (w/v) dodecyl maltoside. The cell suspension was sonicated and separated into soluble and insoluble fractions by centrifugation at 16100 × *g*.

### Blue-Native Polyacrylamide Gel Electrophoresis

Unsealed mitochondrial membranes were isolated as published (17), with minor modifications. Briefly, *Y. lipolytica* cells from 0.5 l overnight culture were harvested by centrifugation at 3500 × *g*. Pellet weights were typically between 2–8 g. Cells were washed with dH_2_0. Per gram cells, 2 g fine glass beads, 2 mm PMSF and 2 ml ice cold mito-membrane buffer (0.6 M sucrose, 20 mm MOPS–NaOH, pH7.5, 1 mm EDTA) were added. Cells were disrupted by 15 rounds of 1 min vortexing and 1 min incubation in ice. Differential centrifugation was performed at 3500 × *g* for 10 min and 40000 × *g* for 120 min at 4°C. Pellets were resuspended in mito-membrane buffer and stored at -80°C. For BN-PAGE, unsealed mitochondrial membranes were mixed with solubilization buffer [750 mm aminocaproic acid, 50 mm Bis-Tris–HCl pH 7.0, 0.5 mm EDTA, 1 % (w/v) dodecyl maltoside] and incubated on ice for 5 min followed by centrifugation at 16100 × *g*, at 4°C, for 10 min. The supernatant containing solubilized membrane proteins were diluted to 0.25 μg μl^-1^ protein (Bradford assay) with solubilization buffer and 0.25% (w/v) Coomassie G250. Typically, 6.25 μg protein was loaded per lane. 5 μl NativeMark™ was used as a molecular weight marker. All BN-PAGE performed used NativePage™ 4–16% Bis-Tris gels (Invitrogen, Waltham, MA USA), with 1 mm spacers. The anode buffer was 50 mm Bis-Tris–HCl, pH7.0; cathode buffer was 50 mm Tricine, 15 mm Bis-Tris–HCl, pH7.0. Gels were run in a XCell SureLock™ system (Thermo Fisher Scientific, Waltham, MA USA) at 4°C, for the first 45 min at 100 V (max 10 mA) with cathode buffer containing 0.02% (w/v) Coomassie G250, then for ∼2.5 h at 250 V (max 15 mA) with cathode buffer containing 0.002% (w/v) Coomassie G250, until the dye front exited the bottom.

BN gels or PVDF membrane were stained in Coomassie solution [45% (v/v) MeOH and 5% (v/v) acetic acid, 0.05% Coomassie R250] for 1–12 h. Gels were destained using a solution of 45% (v/v) methanol and 5% (v/v) acetic acid.

### Protein blot analysis

Mitochondrial membranes were mixed with Laemmli buffer [2% (w/v) SDS, 125 mm Tris–HCl pH 6.8, 10% (w/v) glycerol, 0.04% (v/v) β-mercaptoethanol, and 0.002% (w/v) bromophenol blue] and separated on a 15% SDS-PAGE gel. Proteins were transferred under semi-dry conditions to nitrocellulose membrane (Protran™). Equal loading and transfer was confirmed using Ponceau S stain. Proteins were labelled with antibodies and detected using secondary horseradish peroxidase-conjugated antibodies and chemiluminescence. Mouse monoclonal antibodies against the NUBM and NUCM subunit complex I were a gift from Volker Zickermann. Rabbit polyclonal antibodies against Sdh2 and Aco1 were raised against recombinant *Saccharomyces cerevisiae* proteins and previously reported (40); antibodies against *Y. lipolytica* Ind1 are described in Bych et al. (8).

Protein was transferred from the BN-PAGE gel to a PVDF membrane (0.2 μm, Millipore™) using wet transfer and BN cathode buffer, for 600 min at 40 mA (maximum voltage 100 V). After transfer, immunolabelling was carried out as described above.

### NADH dehydrogenase assays

BN gels were stained for complex I, as described in Sabar et al. (24). Briefly, BN gels were equilibrated in 0.1 M Tris–HCl pH 7.4 and then incubated with new buffer containing 0.2 mm NADH and 0.1% (w/v) NBT. After the desired staining intensity was reached, the reaction was stopped using a solution of 45% (v/v) MeOH and 5% (v/v) acetic acid. Destaining using a solution of 45% (v/v) MeOH and 5% (v/v) acetic acid was performed for optimal enzyme stain visualization.

NADH:HAR oxidoreductase activity was assayed as described (8). Activity for 25 μg mitochondria was measured in a 1 ml volume of 20 mm HEPES–NaOH pH 8.0, 2 mm NaN_3_, 0.2 mm NADH, 2 mm HAR and 40 μg ml^-1^ alamethicin. Measurements were made in a 1-ml quartz cuvette using a UV/Vis spectrophotometer (Jasco, V-550) and started after addition of HAR.

dNADH:DBQ oxidoreductase activity was measured as dNADH oxidation activity in the presence of the ubiquinone analogue n-decylubiquinone (DBQ) as an electron acceptor. Activity for 50 μg mitochondria was measured in a 1 ml volume of 20 mm MOPS–NaOH pH 7.4, 50 mm NaCl, 2 mm KCN, 0.1 mm dNADH and 0.1 mm DBQ. Measurement started after addition of DBQ. 0.01 mm of the complex I inhibitor Piericidin A was added at the end of the reaction to ensure that dNADH:DBQ oxidoreductase activity is due to complex I.

## Supplementary Material

Supplementary Data
