# Supplementary material for: Pathogenic mutations in NUBPL affect complex I activity and cold tolerance in the yeast model Yarrowia lipolytica
Source: Hum Mol Genet. 2018 Jul 4;27(21):3697–709. doi: 10.1093/hmg/ddy247 (PMC6196649; doi:10.1093/hmg/ddy247)
Supplement: Supplementary Data [file ddy247_supp.zip › Supplemental Tables.pdf]

Table S1. Complex I activities in mitochondrial membranes from *Y. lipolytica* Ind1 variants.

Variant	NADH:HAR activity			dNADH:DBQ activity		
	U mg^{-1}	p-value	% of WT	U mg^{-1}	p-value	% of WT
Ind1	1.07 ± 0.06	-	100	0.47 ± 0.02	-	100
e.v.	0.45 ± 0.08	p= 0.003 (**)	42	0.20 ± 0.02	p< 0.001 (**)	43
N271QfsX31	0.37 ± 0.03	p< 0.001 (**)	34	0.17 ± 0.02	p< 0.001 (**)	36
L102P	0.51 ± 0.09	p= 0.007 (**)	48	0.15 ± 0.02	p< 0.001 (**)	32
D103Y	0.31 ± 0.01	p= 0.006 (**)	29	0.18 ± 0.02	p< 0.001 (**)	39
G136D	0.79 ± 0.11	p= 0.088	74	0.37 ± 0.04	p= 0.084	79
L191F	0.69 ± 0.09	p= 0.024 (*)	65	0.28 ± 0.02	p= 0.002 (**)	59
G285C	0.91 ± 0.10	p= 0.222	84	0.31 ± 0.06	p= 0.056	65
*nucm*Δ + *NUCM*^{Y144F}	-	-	-	0.04 ± 0.01	p< 0.001 (**)	9

Table S2. List of primers

Primer	Purpose		5'-3' sequence
AM1	Cloning of *IND1*		TGGCCATGCTTCTACACCCTCC
AM2			TGCTGCCGTCGCTGGTGCCTTT
AM27	Ind1 protein expression		GGCCATGGAAAACCCCCTGGGTATC
AM28			GGCTCGAGCTATTTTTCAAATTGAGG
KB86	Sequencing of *IND1* clones		CTGCAGGTACACCCAACTGCTCAGC
KB87			GGCATATGGAAAACCCCCTGGGTATC
	Site-directed mutagenesis: Amino acid	Nucleotide	
L102P-F	L102P	T305>C	CTGAGGGTAGGACTCCCCGATGTGGACATCTTC
L102P-R			GAAGATGTCCACATCGGGGAGTCCTACCCTCAG
D103Y-F	D103Y	G307>T	CCGAAGATGTCCACATAGAGGAGTCCTACCCTC
D103Y-R			GAGGGTAGGACTCCTCTATGTGGACATCTTCGG
G136D-F	G136D	TG407-408>AT	CCCATGCTCATGACCTGTATATCGAATTTCGACATTGGAATC
G136D-R			GATTCCAATGTCGAAATTCGATATACAGGTCATGAGCATGGG
L191F-F	L191F	C571>T, G573>C	AGTTTGGGCAATAGTGAATTGAACGTCTCCCGTTCCGG
L191F-R			CCGGAACGGGAGACGTTCAATTCACTATTGCCCAAACT
G285C-F	G285C	G853T	GCTCACAGTCAGACAAGTGTGTTCCTGTTGCTGTG
G285C-R			CACAGCAACAGGAACACACTTGTCTGACTGTGAGC
